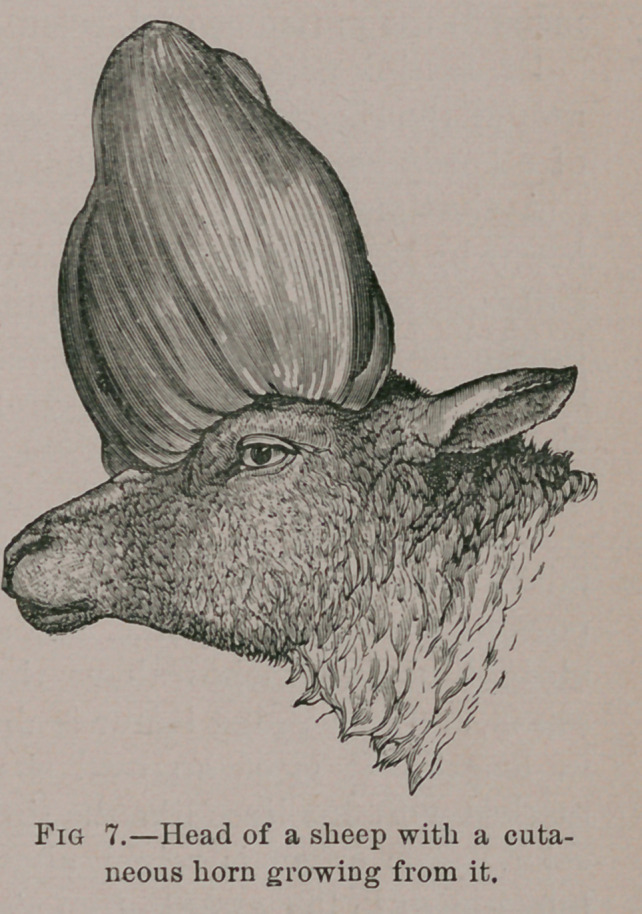# A Comparative Study of Sebaceous Cysts and Cutaneous Horns

**Published:** 1887-01

**Authors:** John Bland Sutton

**Affiliations:** Sir Erasmus Wilson Lecturer, Royal College of Surgeons, Assistant Surgeon and Demonstrator of Anatomy, Middlesex Hospital, London


### Art. II.—A COMPARATIVE STUDY OF SEBACEOUS CYSTS AND CUTANEOUS HORNS.

BY JOHN BLAND SUTTON, F. R. C. S.

*Sir Erasmus Wilson Lecturer, Royal College of Surgeons, Assistant Surgeon and Demonstrator of Anatomy, Middlesex Hospital, London.*

The various structures which arise as differentiations of the integument, and usually included under the term of dermal appendages, are of two distinct kinds: (1) solid structures which are of use in defending, supporting and giving definite form to the organism; (2) glandular organs which are of service in virtue of the secretions they furnish.

To the first group belong such things as hair, nails, claws, hoofs, horns, teeth, bristles, feathers, &c., and to the second, sebaceous glands, their various modifications, sudoriparous glands and the like.

It is to sebaceous glands, in their normal and pathological conditions, that attention will be more especially directed in the present communication.

Like hair, sweat-glands and enamel, sebaceous glands originally appear in the embryo as solid downgrowths or diverticula from the deeper layers of the epiblast. The central portion becomes hollow and receives the secretion provided by the multiplication and subsequent degeneration of the cells lining the walls of the recess.

In man these glands are found wherever there are hairs and the diverticula from which hairs are produced; they sprout from the epidermal cells of the external layer of the root-sheath of the hair. By subsequent branchings from the original downgrowth the more complex varieties of sebaceous glands arise. In very small glands only two or three, or rarely one, recess may be present, in large ones as many as twenty. Their size has no relation to the magnitude of the hair. The fine downy hairs on the nose have very large glands. Klein* points out that the lanugo possesses very large glands, the fine hair being situated, as it were, in the duct of the sebaceous follicle.

In a typical gland the recesses or alveoli possess a limiting membrane, supporting polyhedral cells with oval or spherical nuclei. The central cells are also of this shape and contain minute oil globules.

When the gland is active the cells near the limiting membrane multiply and the product known as *sebum* is forced toward the duct and squeezed, by the gradual accumulation behind, into the neck and mouth of the hair follicle. The duct of the gland is lined with stratified epithelium continuous with the outer root-sheath. Very frequently it happens that the mouth of the duct becomes obstructed whilst secretion continues within the alveoli. The organ thus becomes distended with the outcome of its own activity, and a retention cyst is the result.

Sebaceous cysts of this character occur wherever the glands exist and may vary in size from a pin's head to that of an orange. Their walls may be thin and pliant, or laminated, thick and hard. In the first condition they are formed of thin connective, in the second case composed of tough fibrous tissue. The inner wall of the cyst is lined with squamous epithelium, which may be shed into the cyst in successive layers, giving it a laminated appearance. The contents of sebaceous cysts are usually epithelial scales, granular fatty matter and flakes of cholesterine.

---

* Elements of Histology.

In man sebaceous cysts are far more common on the scalp, where indeed they are often multiple, than in any other situation of the body. They may occur on the back, scrotum and perinæum; in the last named situation I have seen one attain the dimensions of an orange.

They arise in the external auditory meatus, and Toynbee, who has carefully investigated these cases, states that they are found chiefly at the inner part of the meatus close to the membrana tympani. In size they vary from a millet-seed to a large nut, and produce by their pressure enormous distension of the meatus and absorption of the petrous bone to such an extent as to open up a communication with the mastoid cells and even the cranial cavity.

The gradual growth of a sebaceous cyst often leads to very extensive absorption of the underlying structures. Pressure effects of this character are, as a rule, best studied in the cranium. Most pathological museums possess one or more specimens, generally of the skull cap, illustrating this form of absorption. The cyst seems to be lodged in a depression the surfaceof which is perfectly smooth and even.

It is somewhat remarkable that when a bone is exposed to gradual but continuous pressure from without, the osseous tissue is absorbed in such a way that the external table remains perfectly smooth and the cancellous tissue is not exposed. I have collected a number of instances in illustration of this fact. It is also true in a measure of other structures. Once I saw the liver of a deer affected with hydatids, in which a very large cyst had pressed upon the anterior surface of the organ, forming a recess nearly an inch deep, yet the peritoneum covering the depression was intact.

There is a remarkable abnornal condition to which the skin of man is liable, known by the name of Xanthelasma. The disease was originally described by Addison and Gull under the name of Vitiligoidea. There are two forms of the disease: (1) a local affection commonly found near the eyelids and called in consequence *Xanthelasma palpebrarum;* (2) a more general form often associated with jaundice and known as multiple Xanthoma.

The first variety generally appears on the skin near the inner canthus, usually involves a portion of the upper and lower eyelid and is remarkably symmetrical. The patches are as a rule flat, smooth, slightly raised and sharply defined; in color lemon-yellow, buff or orange, resembling very closely chamois leather. Histologically they consist of a fibro-cellular growth, infiltrated with a yellowish oil, which may be found in and around the cells. To this oil is due the yellow color so characteristic of the disease. There are good reasons for believing that the affection is really due to pigmentary changes in the sebaceous glands. In support of this view let me tender the following instructive evidence.

Over the coccygx of most birds is a structure referred to as the uropygial gland, which is nothing else than a large sebaceous gland from which the bird derives ointment for its feathers.

If this oil-gland be examined in the Ground Hornbill, *Bucorvus Abyssinicus,* we shall verify the following points. The orifices of the gland are surrounded by a tuft of fine feathers resembling the point of a camel's-hair brush. If the finger be rubbed over this tuft it will be colored yellow. The beak of the bird is of a beautiful orange-yellow color which also stains the finger when rubbed lightly; the yellow feathers have the same effect. A section of the gland shows it to be of a deep orange color, which easily dissolves out in alcohol and in glycerine. Structurally it resembles the sebaceous variety, the pigment is contained in the cells. We have here an excellent physiological type of Xanthelasma. I know of no other example of this remarkable condition of the uropygial gland. In Plate I, Fig. 1, is a drawing showing the general arrangement and disposition of the lobes.

With regard to Xanthelasma, which has now a somewhat extensive literature, I cannot do better than refer the reader to Mr. Jonathan Hutchinson's paper in the Medico Chir. Trans., vol. 54, p. 171, for some excellent drawings of the morbid appearances.

Sebaceous cysts are liable to secondary changes of various kinds. They may be irritated, inflame and suppurate, thus becoming transformed into abscesses as a consequence. This is usually curative. The cyst may burst its capsule as a result of inflammation, the sebaceous matter becomes exposed, putrifies and gives off an odor usually described by the patient as resembling that of a "putrid red herring." The edges of the everted cyst inflame, granulations arise, the margins become elevated and the mass assumes the superficial characters of an epithelioma, for which it is often mistaken even by observers of experience, but the offensive smell is very characteristic. Microscopically it is difficult to draw the distinction, but clinically the difference is decided. The accompanying drawing, Plate I, Fig. 2, represents a case of this kind, which arose in connection with a sebaceous cyst situated immediately over the parotid gland, a not uncommon situation for sebaceous cysts.

Among other rare secondary changes, such cysts are liable to become calcified; of this I have up to the present time seen only one example; it seemed that the calcareous matter was deposited in the *debris* contained in the cyst.

Warts have been known to develop from the lining membrane and project into the cavity of such cysts, but they are rare.

We must consider a curious condition found associated with sebaceous cysts, and one which I propose to deal with very fully in this article, so as to make it the basis of an explanation of some singular structures in animals.

The contents of a sebaceous cyst may burst through the capsule and, becoming exposed to the air, dry, assume a brownish-black color and become very hard. The mass is composed of epidermal scales which in consequence of the exposure resembles horn in appearance and consistence. If the dried mass is allowed to remain, growth continues at the base until at length a cutaneous horn is produced which may be many inches in length.

The most elaborate collection of cases illustrating this singular condition is to be found in a small work by Dr. Hermann Lebert, entitled "Ueber Keratose oder die durch Bildung von Hornsubstanz erzeugten Krankheiten" (Breslau, 1864). This writer gives an account of no less than one hundred and nine cases, with full references, the earliest dating from the year 1300. The horns were found on the scalp, temples, forehead, eyelids, nose, lips, cheeks, shoulders, arm, elbow, thighs, legs, knee, toes, axilla, thorax, buttock, loin, penis and scrotum. In length they varied from a fraction of an inch to as much as ten or twelve inches, and in circumference some of them measured eight inches. The majority of these cutaneous horns occurred on the head.

An excellent account of human horns is furnished by Sir Erasmus Wilson in his well-known work on "Diseases of the Skin," 5th ed., p. 653. Besides furnishing details of some good examples of these abnormal appendages a brief but interesting resumé of some of the more striking cases is given. The Transactions of the Pathological Society of London contain accounts of many curious examples of cutaneous horns, including one which grew from the prepuce of the clitoris.

Fig. 1.—Cutaneous horns on the face of a woman. The one on the forehead is five inches long.

Elderly females, especially those who are over-cautious lest too much clean water should reach their skin, seem to regard horns with a kind of veneration. But this is not confined to women, for a male patient, who had two horns on his prepuce was terribly alarmed when I suddenly detached them, for a surgeon had warned him that his life depended on their retention. In Fig. 1 two cutaneous horns are represented; the drawing was made from a model in the Museum of the Royal College of Surgeons, London. In this case the abnormal mass is of unusual length.

Leaving man and extending our inquiries to lower animals, we shall find that sebaceous cysts and their consequences are by no means confined to him. They may occur in horses, dogs, sheep, oxen, and birds.

The Museum of the Royal College of Surgeons, London, possesses specimens taken from the head of a partridge, neck of a fowl, wings and legs of wood pigeons, and the head of a blackbird. It is usual to believe that in birds sebaceous glands are wanting, except in the case of the one over the coccygx, known as the oil or uropygial gland, which is especially developed in water fowl and serves as a store of ointment in which the bird dips its beak and anoints the feathers in the act known as preening. It is a very significant fact that every known bird never has its neck shorter than its trunk; that is to say, it is always of sufficient length to allow the bird to reach the oil gland. This structure is not invariably present, for the struthious birds, some of the *Columbæ*, and others, lack an oil gland. In the pigeon it is bilobed, of a whitish color, and a quarter of an inch in length. A duct which is directed backwards has its orifice indicated by a papilla. Such an oil gland as this, is described as being nude. In others it is surrounded by a circlet of small feathers and is then said to be tufted. The majority of birds have two ducts to this gland. In the Hornbill, as before mentioned, the gland is of a deep orange-yellow color. The presence of sebaceous cysts on the wings of the birds mentioned in the preceding list is sufficient to show that the glands in question must exist in the integument on the inner or ventral aspect of the wings.

A typical example of the affection has recently come under my observation in a cockateel, *Calopsitta novæ-hollandiæ.* In this pretty bird a sebaceous cyst had developed on the under surface of each wing. The swellings were symmetrical in size and position. For a drawing of the specimen (which is now preserved in the Museum of the Royal College), the Proc. Zoological Society, London, for 1885, should be consulted.

I have observed undoubted sebaceous cysts not only on the heads of birds, but also of large size in their pectoral region, and the accompanying drawing, taken from the head of a bird in the Hunterian Museum, shows a cyst in connection with the eyelid.

Not merely is there agreement in structure between the sebaceous cysts of birds and animals with those of man, but

FIG. 2.—A sebaceous cyst with laminated contents in connection with the eyelid of a bird.

they exhibit precisely the same tendency to form cutaneous horns.

As a typical case the following account and drawing of a mule canary will be given. The bird (a cross between a linnet and a canary) is preserved in the Museum of the Royal College of Surgeons, London. It had a sebaceous cyst on the left wing. From this wing there sprouted a horny growth which when removed recurred each year for five years. In the preparation, as it stands in the museum, two of the horns detached are shown in the bottle, but the artist has succeeded in representing the wing with the horn as it appeared during life.

FIG. 3.—The wing of a mule canary with a cutaneous horn growing from it.

As far as my inquiries have extended the existence of such cutaneous horns are by no means uncommon in birds confined in cages.

In the same museum is the head of a cock with one of these horny growths sprouting from the external auditory meatus, as shown in Fig. 4.

This specimen is interesting in connection with Toynbee's observations previously mentioned of the occurrence of sebaceous cysts in the meatus near the membrana tympani.

FIG. 4.—Head of a cock with a cutaneous horn, 1½ inches in length, projecting from the external auditory meatus.

Hermann Lebert, in the work previously referred to, mentions instances of cutaneous horns growing from the neck of an old hen, the head of a parrot, and in one case on a greenfinch.

He gives references to similar horns in sheep, he-goats, horses, nose of a ram, hare, cows, dogs, and states that Malpighi described one growing from the neck of an ox, ten finger-breadths in length and eight in circumference at the base.

Everard Home, in 1791, contributed an interesting paper to the Phil. Trans., entitled "Observations on Certain Horny Excrescences of the Human Body." After referring to numerous well-marked and extraordinary cases of horny growths occurring in human beings, he describes the following case in a footnote:

A sheep, about four years old, had a large horn, three feet long, growing on its flank. It had no connection with bone, and appeared only to be attached to the external skin. It dropped off in consequence of its weight having produced ulceration of the soft parts to which it adhered. On examining it there was a fleshy substance, several inches long, of a fibrous texture, filling up its cavity, on which the horn had been formed.

In the Teratological collection of the Royal College of Surgeons there is a horn three feet five inches in length and eleven inches in its greatest circumference, said to have grown on the flank of a ram; preserved in a jar near it is the soft core of the same exactly corresponding to Home's description. The specimen is labelled *Hunterian*, and I have no doubt it is the one referred to above. Its shape and general appearance may be inferred from Fig. 5; *A* is the core or fleshy substance to which Home refers.

A

Fig. 5.—Cutaneous horn growing from the flank of a ram; three feet five inches in length and eleven inches in girth.

In the same collection are the two following: (1) A Hunterian specimen described as "the head of a cow, with a very large horn-like appendage growing from the forehead immediately between the eyes. Fig. 6.

A glance at the specimen is sufficient to assure one that it is really a cutaneous horn. The view is confirmed by the examination of some pieces of tissue preserved in spirit and described as portions of the core of this appendage. Their laminated arrangement and general appearance remove all doubt.

The second specimen is the head of a sheep with what looks like an enormous casque growing from its forehead and gives it an appearance not unlike a Cassowary. Fig. 7. It is described as coming from Mr. Swan's collection in

Fig. 6.—Head of a cow with a cutaneous horn growing from the forehead. (Hunterian.)

1871. The adventitious mass is of the same nature as that on the head of the cow.

These specimens from birds, sheep and cows are clearly pathological in their nature and of the same character as human horns. We must now consider some instances of similar structures occurring normally, which shall serve as "physiological types." It is rather remarkable that we need not venture beyond the quadrumana to find the parts in question, and the following observations made upon lemurs serve to illustrate the question in an admirable manner. They are of increased value in that they are additions to our stock of knowledge concerning dermal organs.

Fig 7.—Head of a sheep with a cutaneous horn growing from it.

In 1884 Mr. F. E. Beddard* drew attention in a paper on *Hapalemur griseus* to a curious structure found on the arm of this lemur.

On the inner side of the fore arm close to the wrist is an oval patch of spine-like processes, about one inch long and one-third of an inch broad in the middle, which is shown in the accompanying drawing (Plate I, Fig. 3). These spines are longest in the middle portion of the patch, and decrease in length towards both extremities. Examined with a hand lens they present the appearance of being composed of a number of fine threads closely bound together; the extremity of the spines is blunt, and the longer ones are somewhat curved and overlap each other. The patch of integument which bears these spines is sharply marked off from the surrounding integument, and no transitional forms between the hairs of the general body-surface and these peculiar spines could be observed.

When the skin of the arm was removed an oval gland of the size and shape of an almond corresponded to this patch of

* Proc. Zoo. Soc. London. P. 393. 1884.

spines on both arms, but Mr. Beddard was unable to ascertain the existence of a duct in connection with the gland.

It has since been ascertained that this patch is present in the two sexes, but in the female the spines are replaced by hairs, but quite distinct from the rest of the integument of the arm. These facts were furnished to Mr. Beddard by Prof. A. Milne-Edwards and Dr. Jentink after an examination of specimens in the museums of Leyden and Paris.

Dr. Jentink pointed out a somewhat similar structure on the arm of the ring-tailed lemur, *Lemur catta*, which has the form of a horny outgrowth somewhat like the spur of a cock.

At the time Mr. Beddard was engaged in dissecting the Hapalemur he kindly afforded me every facility for examining the structure on the arm of this animal, and the likeness of the individual spines to cutaneous horns was very striking. There is little room for doubt that the patch of spines is the result of the hardening of the secretion poured out from the underlying gland.

We must now turn to the structure on the arm of the ring-tailed lemur, *Lemur catta*, regarded by Dr. Jentink as being possibly a climbing-organ. The structure in question is situated about two inches above the wrist joint, on the flexor aspect, and in a young lemur is about three-eighths of an inch in length. It is of an oval shape, soft, compressible, and marked with fine lines like the tip of the finger, and of a black color. The organ is raised above the general level of the integument to the extent of an eighth of an inch. Its major axis lies in the long axis of the limb, and it is continuous with the palm of the hand by a narrow strip of black hairless skin. The organ is present in the male and female. In older lemurs, a hard callous projection is found on the inner side of this body, recalling in every respect the curious spur or wart-like structure which exists on the inner side of the fore and hind leg of the horse and over the fetlock.

This spur or projection in *Lemur catta* resembles, on a large scale, the spines on the arm of *Hapalemur*, and I have no doubt that they both arise in the same way, viz., by exposure and subsequent hardening of the secretion peculiar to the gland.

I had the good fortune to possess among my stores a fœtal *Lemur catta*, and on the forearm, in the situation of the comb-like structure, a few long hairs exist, as in Plate I, Fig. 4; on detaching the integument a glandular looking patch of the bigness of a millet-seed was found. Microscopical examination proved it to be formed of a cluster of glands resembling the sudoriparous variety in structure and opening by long ducts on to the integument, and apparently unconnected with the long hairs which indicate the spot.

I hope, when more material has come into my possession, to be able to give a full account of this structure; but the curious spur of hardened secretion which forms around its orifice brings it into the category of cutaneous horns.

Apart from their general interest, the occurrence of cutaneous horns in man has a special value to the scientific pathologist. For a long time I have been engaged in analyzing structural aberrations, particularly those which are referred to as repetitions of "physiological types"; for instance, the comb of a cock structurally resembles a nævus; the ischial callosities of a baboon, a corn; the cutaneous horns of men, the nasal horn of a rhinoceros, as Hunter was aware; a myxoma repeats the structural peculiarities of the vitreous humour or the structure of the "jelly" in the umbilical cord, and so on.

With regard to cutaneous horns the question admits of two interpretations. Their occurrence in man may be regarded as atavism or reversion to a former condition, which is of normal occurrence in some animals, *e. g.*, lemur, Hapalemur, &c., or it is purely pathological. If the phenomenon is pathological then the warts on the legs of the horse and zebra (see Plate I, Fig. 5) are to be regarded as pathological productions, which have been handed down until they became race-peculiarities.

The more I study the question the more I see that these two causes have been at work, and it is often difficult to decide whether a given structure is atavistic in its nature or a transmitted pathological production.

In addition to sebaceous cysts arising from obstruction to the excretory duct of the gland there are two other varieties which, on the present occasion, must be mentioned only, their full consideration being reserved for some future occasion.

The sebaceous cysts arising in the neck below the deep fascia, and supposed to originate in connection with unobliterated branchial clefts, differ not only in their ætiology but in their nature from the cysts we have been considering. This is also true of similar cysts in the tongue and a few in the iris. Some forms of sebaceous cyst of the iris, and others occurring in the fingers, have been very clearly associated with antecedent injury and are to be attributed to epithelial scales on the one hand, and conjunctival cells or eyelashes on the other, becoming transplanted into deeper structures.

These varieties have never been known to produce horns and are foreign to the subject we have been considering.

NOTE.—Since this was written I have found that a cluster of glands underlie the raised oval patch in the adult of *L. catta.* In other Lemurs, as *L. macaco* and *Chirogaleus coquereli*, the gland is present and indicated by a few long black hairs as in the fœtal *L. catta.*

EXPLANATION OF PLATE.

Fig. 1. The uropygial gland of a hornbill, showing the yellow pigment.
2. Fungating sebaceous cyst in the parotid region of a man.
3. Spiny patch on the forearm of *Hapalemur griseus* (after Beddard).
4. Hand and forearm of a fœtal lemur, *Lemur catta*, to show the gland with its long hairs.
5. Foreleg of horse to show the wart.

## Figures and Tables

**Fig. 1. f1:**
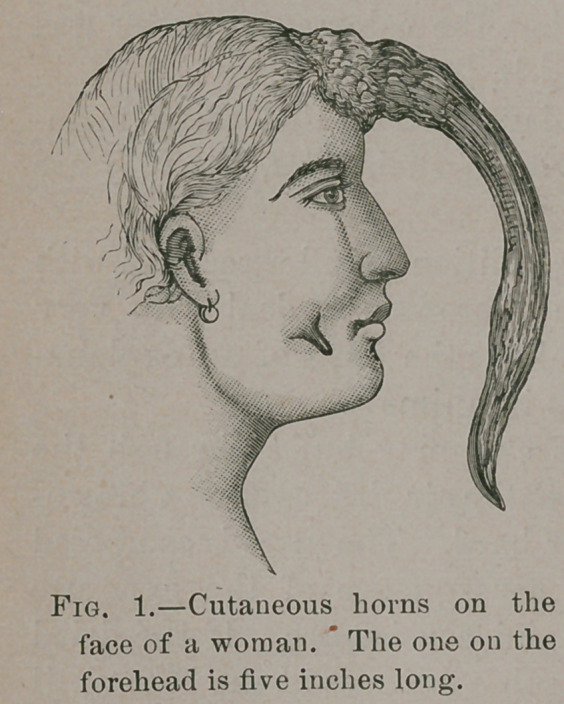


**Fig. 2. f2:**
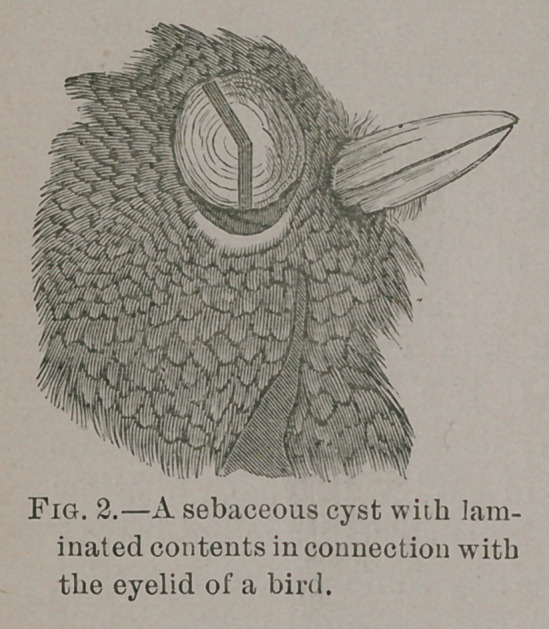


**Fig. 3. f3:**
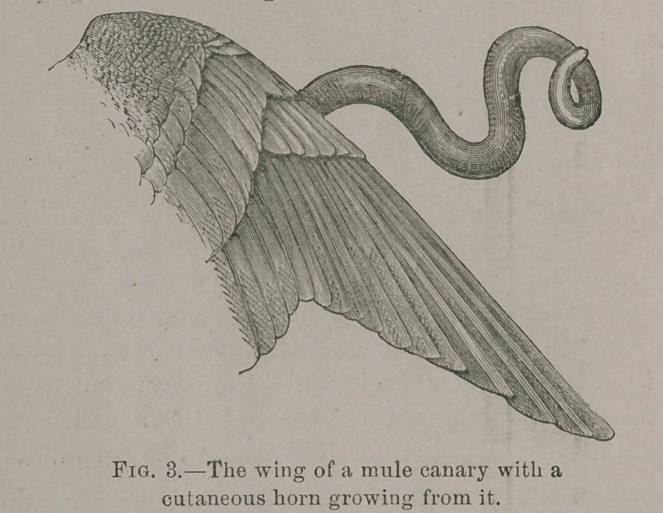


**Fig. 4. f4:**
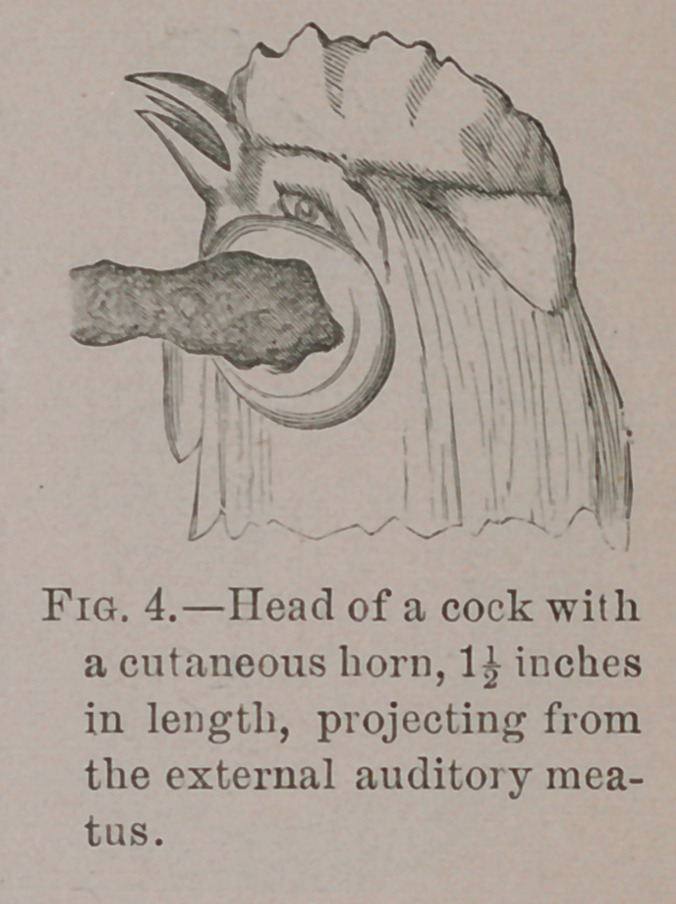


**Fig. 5. f5:**
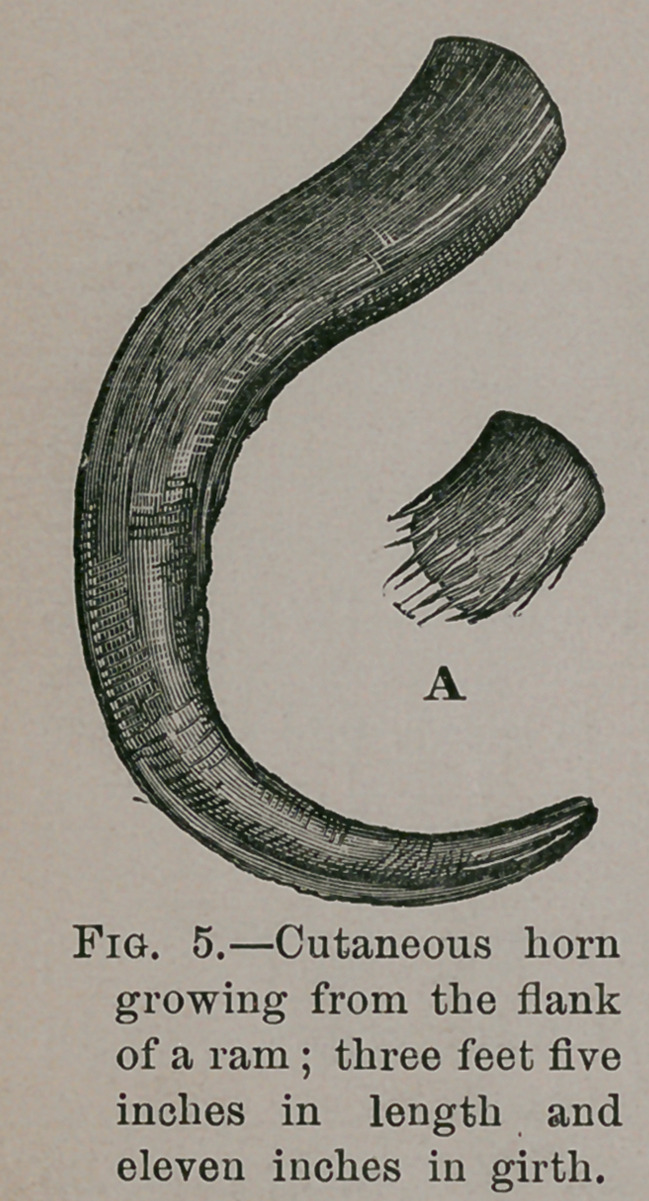


**Fig. 6. f6:**
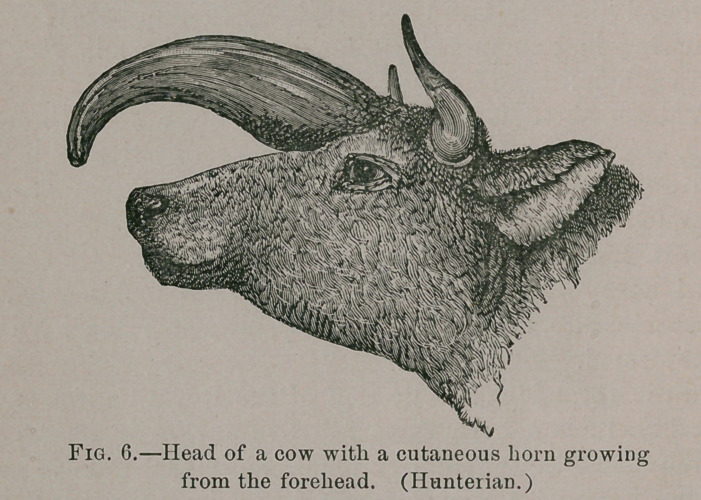


**Fig 7. f7:**